# Quality indicators for palliative care for older people: An umbrella review

**DOI:** 10.1177/02692163251403422

**Published:** 2025-12-29

**Authors:** Amy Hutchison, Amy Spooner, Deborah Parker, Patsy Yates

**Affiliations:** 1 Queensland University of Technology (QUT), Centre for Healthcare Transformation, Faculty of Health, Brisbane, Queensland, Australia; 2 Queensland Health, Metro North Health, Older Persons Emergency Network, Brisbane, Queensland, Australia; 3 University of Technology Sydney (UTS), School of Nursing and Midwifery, Faculty of Health, Ultimo, New South Wales, Australia

**Keywords:** palliative care, ageing, quality indicators, health care, quality of health care

## Abstract

**Background:**

Internationally, the ageing population is driving increased demand for palliative care across primary, aged, and healthcare sectors. Quality indicators for palliative care have been extensively researched, with systematic reviews synthesising these indicators for older populations across various care settings.

**Aim:**

To identify a comprehensive set of quality indicators for palliative care for older people.

**Design:**

A research protocol was developed following Joanna Briggs Institute’s umbrella review methodology and registered with PROSPERO. Included reviews were assessed using the Joanna Briggs Institute’s Critical Appraisal Instrument for Systematic Reviews and Research Synthesis. Identified quality indicators were mapped to Donabedian’s quality model (structure, process, outcome) and aligned with the Australian Commission on Safety and Quality’s essential elements for safe, high-quality end-of-life care.

**Data sources:**

MEDLINE, CINAHL, EMBASE, Scopus, and Cochrane were searched from January 2000 to May 2025.

**Results:**

A total of 1272 records were identified, after removing duplicates 719 were screened by title and for eligibility. Four articles met the criteria, yielding 1071 quality indicators, of which 658 were unique. Among these, 402 (61%) related to the process of care, 179 (27%) to care outcomes, and 77 (12%) to structure.

**Conclusion:**

This review highlights a critical gap in quality indicators related to structure, including the physical and organisational settings of healthcare delivery. Indicators often emphasised a biomedical approach, overlooking the psychological, social, cultural, and spiritual aspects essential to high-quality palliative care. Further work is needed to develop a comprehensive, practical set of quality indicators that can be used across care settings.

**What is already known on this topic?**

- Quality indicators for palliative care have been extensively researched in different populations and settings.
- A comprehensive suite of palliative care quality indicators for older people is required to inform policy and practice.

**What this paper adds**

- This review identified 658 unique quality indicators for palliative care for older people of which 56 indicators required a person or proxy rating, 388 indicators could be derived from healthcare records, and 214 indicators related directly to service or organisational aspects.
- There was a clear lack of quality indicators which relate to the structures of care, demonstrating an underrepresentation of the influence of organisational processes in quality outcomes.
- Indicators often emphasised a biomedical approach, overlooking the psychological, social, cultural, and spiritual aspects essential to high-quality palliative care.

**Implications for practice, theory or policy**

- There is a need for a more refined suite of indicators to be tested across diverse cultural, geographic and healthcare settings.
- This refined suite can then be used by health and aged care services to assess the quality of care they provide and identify performance gaps to target in quality improvement initiatives.

## Introduction

The global population is ageing, and by 2030 it is projected that one in six people will be aged 60 years or over.^1^ As the ageing population grapples with multiple chronic comorbidities, there is an increasing demand for palliative care to be delivered across the primary, health and aged care sectors. Growing evidence suggests that palliative care should be implemented early,^2–4^ and its quality should be assessed against evidence-based criteria. However, defining and assessing quality healthcare is complex and subjective. While the concept of care quality is widely referred to, its definition remains elusive, especially in palliative care which is highly contextual and influenced by individual perspectives and attitudes towards care.^5–7^ Understanding what constitutes high-quality palliative care in diverse care settings is essential for shaping organisational policies and ensuring sustainable quality improvements.^8^

Quality indicators are often used to address the complexities in defining and assessing healthcare quality. These indicators are specific, measurable items based on existing evidence or expert consensus, developed for specific populations and conditions.^9–11^ An ideal quality indicator has four distinct characteristics: a quality guideline, a numerator, a denominator and a performance standard.^10^ By using such indicators, health, primary and aged care services can assess performance against evidence-based criteria, identify performance gaps, and implement targeted improvements in care.

Research on quality indicators for palliative care has expanded across various diseases and populations.^12–21^ While several reviews discuss quality indicators which have relevance to different groups of older people,^12,21–23^ an agreed and comprehensive set of quality indicators focussed on palliative care for the older person more broadly is not available. By using an umbrella review approach, quality indicators identified in such reviews could be synthesised to develop an integrated set of quality indicators specific to palliative care for older people. Hence, this umbrella review aims to identify a suite of palliative care quality indicators for older people, addressing this gap. The review uses Donabedian’s framework to categorise identified quality indicators into three domains: structure, process, and outcome.^7^ “Structure” refers to the setting of care, including the availability of resources and organisational attributes such as adequate staffing. “Process” encompasses the actions involved in care delivery, such as diagnosis and treatment, while “outcomes” reflect the effect of care on individuals and often relates to health status and satisfaction.^7^ Moreover, use of Donabedian’s quality model enables a structured assessment of the relationship between quality indicators and their overall effect on care, and as such, the model is commonly used to facilitate discission and provide recommendations at the individual and organisational levels in which care is provided.^12,13,21,22,24^

## Method

The review followed the Joanna Briggs Institute methodology for umbrella reviews.^25^ A research protocol was developed and prospectively registered with PROSPERO (CRD42024542804) and is reported according to the Preferred Reporting Items for Systematic Reviews and Meta-analyses (PRISMA) guidelines.

### Literature Search

A health research librarian was consulted to assist with development of a search strategy for MEDLINE, CINAHL, Embase, Scopus, and the Cochrane database of systematic reviews as detailed in Supplemental File 1. The initial searches were conducted in May 2024, and a secondary search was conducted in May 2025 to identify any additional articles from May 2024 - May 2025. All results were imported into Covidence for review.^26^ Backward citation tracking of reference lists from included articles was also conducted.

### Eligibility Criteria

Studies were included if they^1^ described undertaking a formal review methodology and quality appraisal of the original articles identified in the review, or of the quality indicators of palliative care or end-of-life care identified in the article,^2^ focussed on quality indicators for older people (defined as people aged 65 years or older, or 50 years or older for Aboriginal and Torres Strait Islander peoples) or the review explored how the identified indicators were applicable to older people. Study protocols and systematic approaches to literature reviews were excluded. Data were limited to full text articles published in English from 01 January 2000.

### Screening, data extraction and quality assessment

All abstracts and selected full text articles were assessed against the inclusion criteria by two independent reviewers. Reasons for exclusion of sources at full text were recorded and reported in the review. Data were extracted from papers by a single reviewer and checked by a second reviewer using a data extraction tool. Data extracted from each quality indicator included the numerator, denominator, level of rigour, performance standard and how the indicator was mapped to Donabedian’s three core concepts: structure, processes, and outcomes. Where this was not available from the review article, the original source was retrieved. Each review was assessed by two independent reviewers for methodological quality using the Joanna Briggs Institute Critical Appraisal Instrument for Systematic Reviews and Research Synthesis.^27^ Any disagreements that arose between the reviewers at each stage of the selection, extraction and appraisal process were resolved through discussion, or with an additional reviewer.

Once extracted, each indicator was coded and reviewed to identify duplicates. Indicators which came from the same original source or described the same measure were consolidated to ensure that only unique indicators were analysed. The quality indicators were then mapped to 1 of the 10 essential elements for safe and high-quality end-of-life care National Consensus Statement.^28^ The essential elements were developed for Australian healthcare services which provide palliative care and are suggested to be used as a comprehensive approach to quality palliative care.^28^ The 10 essential elements made up of 5 care processes (recognising end-of-life, person-centred communication and shared decision making, multidisciplinary collaboration and coordination of care, comprehensive care, and responding to concerns) and 5 organisational processes (leadership and governance, support, education and training, care setting, evaluation, audit and feedback, and systems to support high quality care).^28^ A brief definition of each of these elements is supplied in Supplemental File 2. Each indicator was also assessed to determine how it is measured: directly from the person or their proxy, using healthcare records, or from a service level. Data synthesis was completed by one reviewer and was checked by the other reviewers.

## Results

**Figure 1.**
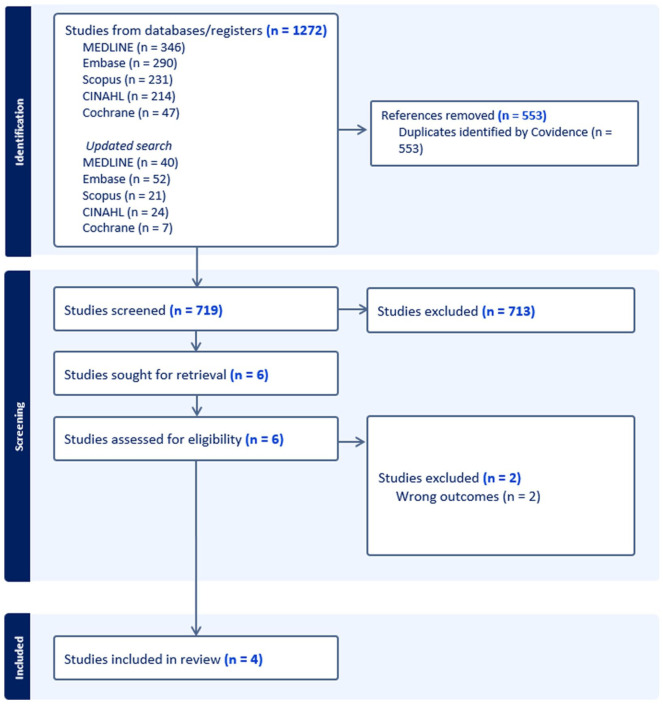
PRIMSA diagram.

The searches were conducted in May 2024, and an updated search in May 2025, with the support of a health research librarian. In total, 1272 records were found from searching. After removing duplicates, 719 articles were screened against the inclusion criteria. Six articles were screened at full text and four articles were identified to meet all criteria. Backwards citation tracking of included articles identified no further articles for review. A PRISMA flow diagram is presented in Figure 1.

### Study characteristics

Of the four articles, two were systematic reviews^12,21^, one was a rapid systematic review,^22^ and one was a systematic review of reviews and guidelines.^23^ Mitchell et al. identified 75 unique quality indicators from 7 different studies. Yorganci et al. identified 976 quality indicators, however, after quality appraisal and shortlisting, only 71 quality indicators were recommended from 7 articles. Amador et al. identified 246 indicators from 19 articles. Karimi-Dehkordi et al. identified 6391 quality indicators, of which 679 related to palliative care, end-of-life care or advance care planning which came from 18 articles and guidelines. The 4 reviews identified quality indicators from a total of 51 original articles.^12,21–23^ Of these, 55% (*n* = 28) were conducted in North America (United States of America: *n* = 19, 37%; Canada: *n* = 9, 18%), 31% (*n* = 16) originated in Europe (The Netherlands: *n* = 7, 14%; Belgium: *n* = 4, 8%; The United Kingdom: *n* = 3, 6%; Finland: *n* = 1, 2%; Switzerland: *n* = 1, 2%), and 14% (*n* = 7) were conducted in Australia.^12,21–23^

Three of the articles reviewed quality indicators for palliative care in aged care,^22^ dementia and older people,^21^ or older people with dementia,^12^ and one article explored quality indicators for older people more broadly.^23^ A summary of the characteristics of each review is presented in Table 1.

**Table 1.** Characteristics of included studies.

Article	Objective/aim	Review type	Journal of publication	Population	Inclusion criteria	Exclusion criteria
Amador et al.,^12^ 2019	To identify and critically evaluate quality indicators for end-of-life care in dementia.	Systematic	Palliative Medicine	No population or setting restrictions were applied in the inclusion/exclusion criteria, however, each indicator was assessed for its relevance to older people dying with or from dementia.	(1) The development and/or characteristics of QIs developed for palliative care (2) numerators and denominators are defined, or can be deduced directly from the description of the QIs, or performance standards given. English translations could be included if available.	(1) editorials, letters to the editor, comments and narrative case reports, (2) indicators focusing on national palliative care policy or the organisation of palliative care at the national level and (3) publications describing the application of existing QIs in clinical practice or reviews of several sets of QIs without any new developments.
Karimi-Dehkordi et al., 2024^23^	Identify existing QIs relevant to the health and care of older adults in community care, continuing care and acute care Categorise the identified QIs based on key attributes to create a taxonomy to facilitate their selection and application	Systematic Review of Reviews and Guidelines	Healthcare	Older adults	Peer-reviewed article, English language, published between 2010 and June 2023. Articles had to include QIs used to measure: (a) health promotion, disease prevention, or population-based/public health; (b) health care in the context of community/primary care, acute care, or continuing care; (c) costs care in acute care or continuing care; or (d) workforce outcomes	Articles which did not collate QIs
Mitchell et al., 2024^22^	To identify population-level indicators of the quality of EOLC for residents of aged care.	Rapid systematic	Archives of Gerontology and Geriatrics	Residents living in an aged care setting (i.e. residential age care, nursing homes, care homes, long-term care facilities).	Each indicator was required to: (i) be developed and/or used for residents of aged care; (ii) specify how the indicator was measured and defined; and (iii) specify how the indicator could be derived from population-based administrative data.	Systematic or other reviews, opinions/editorials, conference abstracts, study protocols or single case reports. Studies that reported on EOLC quality within an ACF using existing EOLC QIs. Articles that focussed on hospice care without being specific to an ACF. Articles and indicators that focussed on advance care planning. Results were limited to English-language articles, published in peer-review journals from 1 January 2000 to 22 November 2022.
Yorganci et al. 2021^21^	(1) Identify and assess the psychometric properties of QIs for the care of older people or people with dementia nearing the end of life and (2) recommend measurable QIs using routinely collected electronic data across care settings.	Systematic	Journal of the American Geriatrics Society	Dementia and/or those who were older.	Studies describing the development, review, and/or testing of QIs for the care of adults with dementia and/or those who were older, and who were nearing the end of life.	Publications reporting the application of existing QIs to clinical practice. Adults younger than 60 years. Papers focussing predominantly on cancer and other disease-specific QIs (e.g. chronic obstructive pulmonary disease and end-stage renal failure). Service performance related QIs.

QIs: quality indicator; EOLC: end-of-life care; ACF: aged care facility.

### Quality of included articles

The quality of each included article was critically appraised, a summary of these ratings is presented in Supplemental File 3. All studies were considered of sufficient quality to be included; however, the main limitation was the variability in the appraisal of articles. Only one review critically appraised each article included within their review.^23^ Two ^21,22^ articles appraised each quality indicator for acceptability, evidence base, definition, feasibility, reliability, and validity, adapted from the works of Henson et al., Rubin et al., Jones et al., and Proctor et al.^17,29–31^ One article^12^ critically appraised the indicators as sets using the Appraisal of Indicators through Research and Evaluation instrument.^32^

### Indicator characteristics

In total, the four articles identified^12,21–23^ 1071 quality indicators, of which 658 are unique. Two articles^21,22^ included quality indicators that could be derived from administrative or electronic datasets. Less than half (*n* = 321, 48%) of the indicators had a numerator and denominator provided and, of these, only 38 (6%) had a benchmark or performance standard provided. All indicators are provided in Supplemental File 4.

### Donabedian’s quality model

All indicators were assessed against Donabedian’s Quality Model and mapped to the essential elements for safe and high-quality end-of-life care National Consensus Statement. Overall, 12% (*n* = 77) of indicators related to a structure of care, 61% (*n* = 402) related to a process of care and 27% (*n* = 179) related to an outcome of care. Of the indicators, 56 (9%) were identified to require the person or their proxy to be directly questioned about palliative care, 388 (59%) could be determined from healthcare records, and 214 (33%) indicators related to the service provider and either did not rely on healthcare records or experiences, or referred to policies or procedures within the service.

### Unique quality indicators requiring person or proxy ratings

A summary of the indicators derived from person or proxy ratings are presented in Table 2.

**Table 2.** Unique quality indicators which required a person or proxy rating (*n* = 56).

Donabedian’s category	Essential element	Number and percentage of indicators	Example indicator
Process (*n* = 24)	Care processes Person-centred communication and shared decision making	11 (45.8%)	Minimal set of Quality Indicators for Palliative Care/Psychosocial aspects of care. Have your professional carers checked how you are feeling?
	Multidisciplinary collaboration and coordination of care	2 (8.3%)	.. Yes, regularly .. Yes, once
	Comprehensive care	10 (41.7%)	.. No
	Organisational processes Evaluation, audit and feedback	1 (4.2%)	Measured by: number of patients who indicated that the caregivers regularly assessed how they were feeling/total number of patients for whom this indicator was measured.
Outcome (*n* = 32)	Care processes Person-centred communication and shared decision making	13 (40.6%)	Care for spiritual well-being of patients: Percentage of relatives who indicate that the patient died peacefully.
	Comprehensive care	12 (37.5%)	If patients die peacefully, this can indicate that in this respect their spiritual needs were met.
	Organisational processes Care setting	1 (3.1%)	Measured by: The number of relatives who indicate that their relative died peacefully/ total number of relatives among whom this quality indicator was measured.
	Evaluation, audit and feedback	6 (18.8%)	

Of the 56 person and proxy rated indicators, 18 (32%) relied on questioning the person experiencing palliative care, 24 (43%) relied on the ratings from family or carers, and 14 (25%) relied on the ratings from healthcare staff.

#### Structure

No indicators that relied on person or proxy ratings of palliative care related to structure of care.

#### Process

Process indicators accounted for 43% (*n* = 24) of quality indicators that required a person or proxy rating. Overall, person-centred communication and shared decision making was the most prominent theme within these indicators (46%, *n* = 11).

#### Outcome

Outcome indicators accounted for 57% (*n* = 32) of person and proxy quality indicators. These indicators were also most commonly mapped to person-centred communication and shared decision making, with 41% (*n* = 13) of indicators in this category.

Within the process and outcome categories, in the person-centred communication and shared decision making element, the quality indicators commonly related to care planning with the person and their family (*n* = 11), family care (*n* = 6), spiritual and psychosocial care (*n* = 7) and communication with the person (*n* = 2). These indicators were framed as a question to be asked directly to the person or their family/carers regarding goals of care and satisfaction with the care provided.

### Unique quality indicators derived from health care records

A summary of the indicators that can be derived from healthcare records are presented in Table 3.

**Table 3.** Unique quality indicators which can be measured from healthcare records (*n* = 388).

Donabedian’s category	Essential element	Number and percentage of indicators	Example indicator
Process (*n* = 254)	*Care processes* Recognising end of life Person-centred communication and shared decision making	1 (0.4%) 53 (20.9%)	Adults in the last days of life, and the people important to them, are given opportunities to discuss, develop and review an individualised care plan.
	Multidisciplinary collaboration and coordination of care Comprehensive care	18 (7.1%) 168 (66.1%)	Proportion of adults recognised as being in the last days of life, and the people important to them, who are given opportunities to discuss and develop an individualised care plan.
	*Organisational processes* Care setting Evaluation, audit and feedback Systems to support high quality care	3 (1.2%) 9 (3.5%) 2 (0.8%)	Measured by: the number in the denominator with care records that show the person who was in the last days of life, and the people important to them, were given opportunities to discuss and develop an individualised care plan/the number of adults recognised as being in the last days of life.
Outcome (*n* = 134)	*Care processes* Recognising end of life Person-centred communication and shared decision making	1 (0.7%) 5 (3.7%)	For patients who screened positive for pain, the percent with improvement within 1 day of screening.
	Multidisciplinary collaboration and coordination of care Comprehensive care	6 (4.5%) 58 (43.3%)	Measured by: Numerator: Number of patients with (0 ⩽ Improvement Time ⩽ 1) and (Improvement = 1; Improvement Time = Second Pain assessment date-Pain assessment date)/ Patients with pain.
	*Organisational processes* Care setting Evaluation, audit and feedback Systems to support high quality care	11 (8.2%) 42 (31.3%) 11 (8.2%)	

#### Structure

No indicators measured from healthcare records related to structure of care.

#### Process

Process indicators accounted for 65% (*n* = 254) of quality indicators measured from a healthcare record. Overall, comprehensive care was the most prominent theme within these process indicators (66%, *n* = 168).

#### Outcome

Outcome indicators accounted for 35% (*n* = 134) of quality indicators derived from healthcare records. These quality indicators were also most commonly mapped to comprehensive care, with 43% (*n* = 58) of outcome indicators within this category. Evaluation, audit and feedback accounted for 31% (*n* = 42) of indicators, and these related to the rate of care transitions, frequency of hospital presentations and timeliness of referrals to other services or settings of care.

Within the comprehensive care domain, across both process and outcome, quality indicators commonly referred to specific aspects of care such as symptom assessment and management (*n* = 104, 46%), medication use (*n* = 47, 21%) and ethical and legal aspects of care (*n* = 30, 13%).

### Unique quality indicators relating to the service level

A summary of the indicators relating directly to the service provider are presented in Table 4.

**Table 4.** Unique quality indicators identified to relate directly to the service level (*n* = 214).

Donabedian’s category	Essential element	Number and percentage of indicators	Example indicator
Structure (*n* = 77)	Care processes Recognising end of life Person-centred communication and shared decision making Multidisciplinary collaboration and coordination of care Comprehensive care	1 (1.3%) 2 (2.6%) 15 (19.5%) 2 (2.6%)	For acute hospitals: They have processes in place to identify the training needs of all workers (registered and unregistered) in the hospital that take into account the four core common requirements for workforce development (communication skills, assessment and care planning, advance care planning, and symptom management) as they apply to end of life care.
	Organisational processes Leadership and governance Support, education and training Care setting Evaluation, audit and feedback Systems to support high quality care	30 (39.0%) 4 (5.2%) 8 (10.4%) 2 (2.6%) 13 (16.9%)	Measured by: Proportion of workers attending educational programmes related to end of life care for registered workers.
Process (*n* = 124)	Care processes Person-centred communication and shared decision making Multidisciplinary collaboration and coordination of care Comprehensive care	5 (4.0%) 13 (10.5%) 73 (58.9%)	Pain: if a cancer patient is admitted to a hospital then there should be screening for the presence or absence of pain.
	Organisational processes Leadership and governance Support, education and training Evaluation, audit and feedback Systems to support high quality care	9 (7.3%) 1 (0.8%) 2 (1.6%) 21 (16.9%)	
Outcome (*n* = 13)	Care processes Comprehensive care	5 (38.5%)	Quality measure for district/community nursing services. They have practical arrangements in place to support those dying at home or in care home.
	Organisational processes Leadership and governance Evaluation, audit and feedback Systems to support high quality care	2 (15.4%) 3 (23.1%) 3 (23.1%)	Measured by: Proportion of cases with equipment, supplies and crisis boxes in place and out of hours sitting services available.

#### Structure

In total, 77 service level quality indicators related to the structures of care were identified, and these focussed on policies, procedures and documentation available in the healthcare setting. Over 70% of the indicators in this category related to organisational processes, such as leadership and governance (*n* = 30, 39%) processes within an organisation.

#### Process

A total of 124 service level process quality indicators were identified, accounting for 58% of the service indicators. Most quality indicators fell under the comprehensive care essential element (*n* = 73, 59%) and related to policies and procedures developed within the service.

#### Outcome

Overall, 6% (*n* = 13) of the service level indicators referred to an outcome of care. Of these, five indicators (39%) were related policies and procedures within a service relating to comprehensive care elements.

## Discussion

This umbrella review identified 658 unique quality indicators relating to palliative care provision for older people, extracted from 4 reviews focussed on older people, aged care and dementia. These quality indicators were diverse and reflected both individual experience of palliative care, and service delivery of palliative care.

### Quality indicators requiring person or proxy ratings

Several indicators required the person, or their proxies, to rate their palliative care experience, reinforcing the importance of person-centred care. These indicators aimed to ensure that the person and their family are appropriately informed of the person’s prognosis and are involved in care planning. Other indicators focussed on the relationship between the person, their family, and healthcare staff, assessing the timeliness of care, support provided by the palliative care team, and the quality of communication between staff, individuals, and families. A positive relationship between the person, their family, and the healthcare team is central to effective palliative care and qualitative studies have demonstrated its importance in supporting wellbeing, maintaining quality of life, and adherence to documented end-of-life wishes.^33,34^ Routine use of person reported outcomes has also been demonstrated to produce a positive effect on these person/provider interactions, symptom identification, monitoring and management, care responsiveness, and satisfaction.^35^ While some may view asking people near end-of-life about their care experience as potentially uncomfortable or burdensome, such conversations can yield valuable insights into care quality as evidenced by the indicators identified in this review. Moreover, they can offer an opportunity for storytelling and meaningful engagement, even within a research context.^36^

### Quality indicators derived from healthcare records

A total of 388 unique quality indicators could be derived from healthcare records. A significant portion of these indicators focussed on comprehensive assessments and symptom management, as well as aligning the care provided with the goals and preferences of the individual. This approach ensures that care is personalised and responsive to the specific needs of each person with the aim of addressing physical and psychosocial aspects of care. The indicators also emphasised the importance of regular assessments of the individual’s condition and preferences, ensuring that care plans are adjusted as necessary to provide optimal support for the older person and their family throughout the palliative care journey. A number of these indicators related to older people with dementia and highlighted the importance of ongoing screening and assessment of their cognition and functional needs, alongside communicating with and involving the person, their family or substitute decision maker in all aspects of care, appropriate to the needs and cognition of the person receiving palliative care. While it is important to emphasise the need for indicators of this nature for people with dementia, the fundamental principles of this type of care are transferrable to all people receiving palliative care; the importance of tailoring care delivery to the needs of the person and involving their family or substitute decision makers to best support the person receiving palliative care.

Indicators derived from healthcare records offer several advantages to health service providers due to their accessibility and independence from staff recall. These attributes make them an appealing tool for auditing purposes, facilitating efficient identification of performance gaps at scale, and as such, palliative care audit tools have been developed with this objective in mind.^37^ While these record-derived indicators and tools are attractive, they present some limitations. Documentation in the healthcare record does not always guarantee that the intervention was delivered effectively or experienced positively by the person. Such data also depend on consistent and accurate reporting of care. Consequently, there is a risk that the presence of a record may not accurately reflect the quality of care delivered.

### Quality indicators relating directly to service level

Overall, 214 quality indicators related directly to the service level, covering the structure, process, and outcome of care. The service provider indicators primarily aimed to evaluate the organisation’s ability to deliver effective palliative care to older people. These indicators commonly referenced the service’s internal policies, staff training and competency, as well as the documentation processes that ensure these policies are being followed. Many of these indicators were categorised under the process of care (*n* = 124, 58%), reflecting the importance of structured care delivery methods, coordination between different healthcare professionals, and adherence to established palliative care guidelines.

Several of these service level indicators related to specific settings of care, namely acute care, residential aged care and community care. Indicators relating to acute care and residential aged care settings emphasised the importance of having timely access and availability of specialist services, processes and policies for admission and transfer of palliative people, and appropriate physical environments to support a dying person and their family, such as private spaces for family meetings, unrestricted family visitation policies, and access to a single room. Indicators relating to community care often described processes related to supporting the person to die at home, such as home visits performed by physicians and other health care staff prior to and after the persons death and ensuring the person had access to end of life equipment at home. It is well documented that most people would prefer to die at home^38–40^ and families and caregivers are increasingly relied upon to care for the dying person by providing assistance with activities of daily living, supporting medical decision making and administering medications,^41^ yet no service level quality indicators described organisational policies, processes or interventions for assessing carers willingness and availability to care for the dying person, and subsequent training of these carers in end of life symptom management, medication administration, and other activities to support a dying person. The ageing population will demand an increased reliance on informal carers to support palliative care delivery in private homes, and as such, health, aged and primary care services should incorporate assessment, education and support tools within their organisational processes to ensure that informal carers are willing to provide end of life care and are supported to do this at a high quality.^42,43^

Together, the person and proxy rated, healthcare record and service level quality indicators provide a multidimensional perspective on palliative care, highlighting the need for both personalised care and organisational structures that support the delivery of high-quality palliative care.

### Structure

Only 77 indicators addressed care structure, all at the service level. Donabedian argues that the structure of a healthcare system influences the processes of that organisation, which in turn leads to positive or negative healthcare quality outcomes.^7^ Evidence suggests that structural components of care such as adequate staffing, appropriately educated staff, continuity of care, and greater access to palliative care equipment contributes to a more positive experience of palliative care, improved palliative care quality, and reduced healthcare expenditure.^44–49^ The importance of structure is not reflected in the indicator set, where only 12% of the total number of quality indicators were assessed as a structure of care. This highlights a significant underrepresentation of the influence of organisational structure in quality outcomes and is consistent with other palliative care quality indicator reviews^13,24^ highlighting potential gaps in practice.

### Process

Most indicators related to processes of care (*n* = 402, 61%), and specifically comprehensive care. These indicators commonly related to medication management, symptom assessment, and diagnostic testing. Indicators of this nature are relatively easy to define, conceptualise and benchmark, and are important for inclusion in palliative care audit tools. However, such indicators reflect a distinctly biomedical approach to care, with indicators that reflect a more holistic approach not as prominent. Indicators which reflect quality in areas of cultural, spiritual, ethical, legal and financial needs are more challenging to conceptualise and define. Few indicators related to care after death, including grief and bereavement of the older persons’ family or caregivers, highlighting the need for future development of indicators in this area. A co-design approach where consumers and providers are jointly involved in the development of such quality indicators would provide valuable insight into what constitutes high quality care from the perspectives of those giving and receiving care in these more complex domains. The integration of consumer voices in healthcare is increasingly recognised as an essential component of organisational improvement,^50^ and studies suggest that consumers and researchers find partnering a positive experience,^50–52^ highlighting the importance of leveraging consumers experiences in healthcare quality decision making. Despite these limitations, the second highest proportion of indicators related to person-centred care and shared decision-making, further reinforcing that a person-centred approach to palliative care is fundamental to high-quality care.^53,54^

### Outcome

Within the outcomes of care category, evaluation, audit and feedback was a prominent indicator type (*n* = 51, 28%), and these related to rates of transfer between facilities, frequency and length of hospitalisation, and timeliness of referrals to services such as hospice care. In addition, care setting made up 7% of the outcome indicators, and these related to the place in which a person died, and the appropriateness of that setting. These indicators jointly highlight that the environment in which a person receives palliative care may impact on the quality and experience of care. Palliative care is recognised as a human right, and as such, should be provided at a high standard for each person regardless of geographical location and care setting.^3^ Available data indicate, however, that outcomes of care can differ between acute care settings, hospices, private homes, and aged care facilities.^55,56^ A number of negative indicators related to hospitalisation, emergency department visits, intensive care stays within the last weeks to months of life, and high rates of transfer to these settings.^12,22^ Some caution is required in interpreting such indicators in this way. Indeed, accessing acute care services to manage reversible illnesses or acute palliative emergencies is good practice in some circumstances. Moreover, while overtreatment in the last days of life can occur if a person is hospitalised,^57,58^ and research suggests that many people prefer to die at home,^39^ recent studies have reported that that home based palliative care can be associated with worse symptom management.^55,59,60^ These poorer outcomes can be attributed to insufficient planning and lack of availability of out-of-hours home care support, even though home based care may offer a more peaceful death and reduce grief for the person’s family or caregivers.^55,59,60^ The outcome quality indicators identified in this review do not always acknowledge the need for older people to die in their place of preference and that these preferences can change over time. They also do not consider the need for informed choice about the effectiveness of services that are available within various settings. Indicators which address how health, aged and primary care sectors work collaboratively to provide timely and reliable access to palliative care across settings are required.

### Variability of included studies and quality indicators

Most indicators lacked the essential components of a quality indicator: a definition, numerator, denominator, and benchmark. Only 6% (*n* = 38) had all four components, highlighting that a number of indicators may be underdeveloped or inappropriate for use. Additionally, there was noticeable variability in the quality appraisal of the indicators. Mitchell et al. and Yorganci et al. identified several indicators from the same source, however, the quality appraisal of some indicators differed despite using the same criteria. While psychometric assessment is an important aspect of the review of quality indicators, the interpretation or application of the assessment may differ between assessors, demonstrating the requirement for quality indicators to be rigorously reviewed and tested upon implementation to ensure that data are collected and interpreted in line with the original intention of the indicator.

Two articles^21,22^ were limited to indicators derived from administrative datasets and as a result, the subjective nature of healthcare needs such as spiritual, religious and cultural aspects of care were not examined. Mitchell et al. also excluded quality indicators relating to advance care planning. Amador et al. and Karimi-Dehkordi et al. however, identified more inclusive sets of indicators which included these concepts, highlighting the importance of conducting this review to devise a comprehensive list of indicators for use in this population. These differing approaches likely contributed to the large variation in number of quality indicators identified in each review. The lack of a consistent approach to defining and operationalising quality indicators in palliative care suggests that current approaches to measuring quality are likely to be inadequate.

### Recommendations

Future research should focus on refining and testing a comprehensive suite of indicators that encompass the three components of healthcare quality: structure, process and outcomes of care. These indicators should reflect both the organisational processes that influence quality of care, and the physical and psychological, social, and spiritual dimensions of care. By testing these indicators, their validity for use in older people can be rigorously evaluated and may be then used by health and aged care services to identify performance gaps in palliative care delivery. Evaluating the effectiveness of these quality indicators in diverse cultural, geographical, and healthcare settings would further enhance their applicability and relevance. Additionally, there is a need for a more standardised, universal approach to appraising quality indicators to ensure consistency in their revaluation. Continuous feedback mechanisms should be established to ensure that quality indicators are regularly reviewed and updated based on emerging evidence and palliative care outcomes. The integration of technology, such as electronic health records and data analytics, should be considered to track and improve the delivery of care in real-time, facilitating better decision-making and communication across care teams.

### Strengths and limitations

Employing a rigorous umbrella review methodology to assess the quality of the included reviews is a strength of this review. This umbrella review synthesises indicators from four systematic reviews that examined different groups of older people to provide a comprehensive list of palliative care quality indicators for this population. This review is limited, however, by the small number of reviews available on this topic. Two studies^21,22^ limited their reviews to quality indicators that could be derived from an administrative or electronic database, which limits the depth of the included indicators. This highlights the need for ongoing research into palliative care quality indicators for older people to be used in evaluation of palliative care outcomes. Umbrella reviews are a rigorous research method, however, there is always a risk that relevant studies have been omitted in the search and review of articles, and that relevant data have been omitted by error in the extraction or appraisal of the included data. This risk was minimised by using a comprehensive search strategy, multiple databases and for the title, abstract and full text screening to have been completed by two reviewers and double checking the data extraction process.

## Conclusion

This umbrella review identified 658 unique palliative care quality indicators for older people. These indicators covered a diverse range of clinical and operational aspects of care; however, it is recognised that they may not encapsulate all aspects of quality of palliative care. There is a need to further develop, refine and test these indicators in a sample population to ensure their accuracy in measuring quality of palliative care for older people and their overall adherence to national standards and guidelines for safe and effective palliative care quality.

## Supplemental Material

sj-docx-1-pmj-10.1177_02692163251403422 – Supplemental material for Quality indicators for palliative care for older people: An umbrella review Supplemental material, sj-docx-1-pmj-10.1177_02692163251403422 for Quality indicators for palliative care for older people: An umbrella review by Amy Hutchison, Amy Spooner, Deborah Parker and Patsy Yates in Palliative Medicine

sj-docx-2-pmj-10.1177_02692163251403422 – Supplemental material for Quality indicators for palliative care for older people: An umbrella review Supplemental material, sj-docx-2-pmj-10.1177_02692163251403422 for Quality indicators for palliative care for older people: An umbrella review by Amy Hutchison, Amy Spooner, Deborah Parker and Patsy Yates in Palliative Medicine

sj-docx-3-pmj-10.1177_02692163251403422 – Supplemental material for Quality indicators for palliative care for older people: An umbrella review Supplemental material, sj-docx-3-pmj-10.1177_02692163251403422 for Quality indicators for palliative care for older people: An umbrella review by Amy Hutchison, Amy Spooner, Deborah Parker and Patsy Yates in Palliative Medicine

sj-xlsx-4-pmj-10.1177_02692163251403422 – Supplemental material for Quality indicators for palliative care for older people: An umbrella review Supplemental material, sj-xlsx-4-pmj-10.1177_02692163251403422 for Quality indicators for palliative care for older people: An umbrella review by Amy Hutchison, Amy Spooner, Deborah Parker and Patsy Yates in Palliative Medicine
