# Supplementary material for: Quality indicators for palliative care for older people: An umbrella review
Source: Palliat Med. 2025 Dec 29;40(3):284–96. doi: 10.1177/02692163251403422 (PMC12936153; doi:10.1177/02692163251403422)
Supplement: sj-docx-1-pmj-10.1177_02692163251403422 – Supplemental material for Quality indicators for palliative care for older people: An umbrella review [file sj-docx-1-pmj-10.1177_02692163251403422.docx]

### Supplementary File 1: Search strategy

**CINAHL (EBSCOhost)**

1 (MH "clinical indicators") OR (MH "quality of care research") OR (MH "quality of health care")

2 AB (“quality indicat*” OR "quality measure*" OR "quality criteri*" OR "quality assessment" OR ((Quality OR performance OR satisf*) AND (indicator* OR criteri* OR assess* OR measur* OR scale OR validat*)))

3 TI (“quality indicat*” OR "quality measure*" OR "quality criteri*" OR "quality assessment" OR ((Quality OR performance OR satisf*) AND (indicator* OR criteri* OR assess* OR measur* OR scale OR validat*)))

4 1 OR 2 OR 3

5 (MH "palliative care") or (MH "palliative care nursing") OR (MH “advance care planning”) OR (MH “attitude to death”) OR (MH “bereavement”) OR (MH “terminal care”) OR (MH “hospice care”) OR (MH “terminally ill patients”) OR (MH “death”)

6 AB (“end of life” OR “end-of-life” OR palliat* OR “end stage” OR terminal* or "late stage" or dying or eol OR hospice* or “advance care plan*” OR “attitude to death” OR “bereavement” OR “life supportive care”)

7 TI (“end of life” OR “end-of-life” OR palliat* OR “end stage” OR terminal* or "late stage" or dying or eol OR hospice* or “advance care plan*” OR “attitude to death” OR “bereavement” OR “life supportive care”)

8 5 OR 6 OR 7

9 (MH “aged”) OR (MH “frail elderly”) OR (MH "aged, 80 and over”) OR (MH "geriatrics”)

10 AB (elder* or geriatric* or octogenarian* or nonagenarian* or gerontol* or “old* age*” or “old* people” or “old* person” or “old* adult” or “old* Australian*” or “old* men” or “old* women” or “old* resident*” or “old* patient*” or “senior citizen*” or “late life” or “oldest old” or “aged 65” or “aged 70” or “aged 75” or “aged 80” or “aged 85”)

11 TI (elder* or geriatric* or octogenarian* or nonagenarian* or gerontol* or “old* age*” or “old* people” or “old* person” or “old* adult” or “old* Australian*” or “old* men” or “old* women” or “old* resident*” or “old* patient*” or “senior citizen*” or “late life” or “oldest old” or “aged 65” or “aged 70” or “aged 75” or “aged 80” or “aged 85”)

12 9 OR 10 OR 11

13 AB ("systematic review*" or "meta analysis" or "meta-analysis" or "scoping review" or “realist review” or “systematic integrative review” or “mapping review” or “critical review” or “qualitative review” or “meta synthesis” or “quantitative review” or "rapid review")

14 TI ("systematic review*" or "meta analysis" or "meta-analysis" or "scoping review" or “realist review” or “systematic integrative review” or “mapping review” or “critical review” or “qualitative review” or “meta synthesis” or “quantitative review” or "rapid review")

15 13 OR 14

16 4 AND 8 AND 12 AND 15

**MEDLINE (EBSCOhost)**

1 (MH "quality indicators, health care") OR (MH "quality of health care")

2 AB (“quality indicat*” OR "quality measure*" OR "quality criteri*" OR "quality assessment" OR ((Quality OR performance OR satisf*) AND (indicator* OR criteri* OR assess* OR measur* OR scale OR validat*)))

3 TI (“quality indicat*” OR "quality measure*" OR "quality criteri*" OR "quality assessment" OR ((Quality OR performance OR satisf*) AND (indicator* OR criteri* OR assess* OR measur* OR scale OR validat*)))

4 1 OR 2 OR 3

5 (MH "palliative care") or (MH "hospice and palliative care nursing") OR (MH “advance care planning”) OR (MH “attitude to death”) OR (MH “bereavement”) OR (MH “terminal care”) OR (MH “hospice care”) OR (MH “terminally ill patients”) OR (MH “death”)

6 AB (“end of life” OR “end-of-life” OR palliat* OR “end stage” OR terminal* or "late stage" or dying or eol OR hospice* or “advance care plan*” OR “attitude to death” OR “bereavement” OR “life supportive care”)

7 TI (“end of life” OR “end-of-life” OR palliat* OR “end stage” OR terminal* or "late stage" or dying or eol OR hospice* or “advance care plan*” OR “attitude to death” OR “bereavement” OR “life supportive care”)

8 5 OR 6 OR 7

9 (MH “aged”) OR (MH “frail elderly”) OR (MH "aged, 80 and over”) OR (MH "geriatrics”)

10 AB (elder* or geriatric* or octogenarian* or nonagenarian* or gerontol* or “old* age*” or “old* people” or “old* person” or “old* adult” or “old* Australian*” or “old* men” or “old* women” or “old* resident*” or “old* patient*” or “senior citizen*” or “late life” or “oldest old” or “aged 65” or “aged 70” or “aged 75” or “aged 80” or “aged 85”)

11 TI (elder* or geriatric* or octogenarian* or nonagenarian* or gerontol* or “old* age*” or “old* people” or “old* person” or “old* adult” or “old* Australian*” or “old* men” or “old* women” or “old* resident*” or “old* patient*” or “senior citizen*” or “late life” or “oldest old” or “aged 65” or “aged 70” or “aged 75” or “aged 80” or “aged 85”)

12 9 OR 10 OR 11

13 AB ("systematic review*" or "meta analysis" or "meta-analysis" or "scoping review" or “realist review” or “systematic integrative review” or “mapping review” or “critical review” or “qualitative review” or “meta synthesis” or “quantitative review” or "rapid review")

14 TI ("systematic review*" or "meta analysis" or "meta-analysis" or "scoping review" or “realist review” or “systematic integrative review” or “mapping review” or “critical review” or “qualitative review” or “meta synthesis” or “quantitative review” or "rapid review")

15 13 OR 14

16 4 AND 8 AND 12 AND 15

**EMBASE**

1 'health care quality'/mj OR 'clinical indicator'/mj

2 (“quality indicat*” OR "quality measure*" OR "quality criteri*" OR "quality assessment" OR ((Quality OR performance OR satisf*) AND (indicator* OR criteri* OR assess* OR measur* OR scale OR validat*))):ab,ti,kw

3 1 or 2

4 'terminal care'/mj OR 'palliative nursing'/mj OR 'palliative treatment'/mj OR 'advance care planning'/mj OR 'attitude to death'/mj OR 'bereavement support'/mj OR 'hospice care'/mj OR 'terminally ill patient'/mj OR 'death'/mj

5 (“end of life” OR “end-of-life” OR palliat* OR “end stage” OR terminal* or "late stage" or dying or eol OR hospice* or “advance care plan*” OR “attitude to death” OR “bereavement” OR “life supportive care”):ab,ti,kw

6 4 or 5

7 'aged'/mj OR 'frail elderly'/mj OR 'very elderly'/mj OR 'geriatrics'/mj

8 (elder* or geriatric* or octogenarian* or nonagenarian* or gerontol* or “old* age*” or “old* people” or “old* person” or “old* adult” or “old* Australian*” or “old* men” or “old* women” or “old* resident*” or “old* patient*” or “senior citizen*” or “late life” or “oldest old” or “aged 65” or “aged 70” or “aged 75” or “aged 80” or “aged 85”):ab,ti,kw

9 7 or 8

10 ("systematic review*" or "meta analysis" or "meta-analysis" or "scoping review" or “realist review” or “systematic integrative review” or “mapping review” or “critical review” or “qualitative review” or “meta synthesis” or “quantitative review” or "rapid review"):ab,ti,kw

11 3 AND 6 AND 9 AND 10

**Scopus**

TITLE-ABS(("quality indicat*" OR "quality measure*" OR "quality criteri*" OR "quality assessment" OR ((Quality OR performance OR satisf*) AND (indicator* OR criteri* OR assess* OR measur* OR scale OR validat*))) AND ("end of life" OR "end-of-life" OR palliat* OR "end stage" OR terminal* or "late stage" or dying or eol OR hospice* or "advance care plan*" OR "attitude to death" OR "bereavement" OR "life supportive care") AND (elder* or geriatric* or octogenarian* or nonagenarian* or gerontol* or "old* age*" or "old* people" or "old* person" or "old* adult" or "old* Australian*" or "old* men" or "old* women" or "old* resident*" or "old* patient*" or "senior citizen*" or "late life" or "oldest old" or "aged 65" or "aged 70" or "aged 75" or "aged 80" or "aged 85") AND ("systematic review*" or "meta analysis" or "meta-analysis" or "scoping review" or "realist review" or "systematic integrative review" or "mapping review" or "critical review" or "qualitative review" or "meta synthesis" or "quantitative review" or "rapid review"))

**Cochrane database of systematic reviews**

1 MeSH descriptor: [Quality of Health Care] this term only

2 (“quality indicator” OR "quality measure" OR "quality criteria" OR "quality assessment" OR ((Quality OR performance OR satisf*) AND (indicator* OR criteri* OR assess* OR measur* OR scale OR validat*))):ti,ab

3 MeSH descriptor: [Palliative Care] this term only

4 (“end of life” OR "end-of-life" OR palliat* OR “end stage” OR terminal* or "late stage" or dying or eol OR hospice* or “advance care plan” OR “attitude to death” OR “bereavement” OR “life supportive care”):ti,ab

5 MeSH descriptor: [Aged] this term only

6 (elder* or geriatric* or octogenarian* or nonagenarian* or gerontol* or “old age” or “old people” or "older people" or “old person” or "older people" or “older adult” or “older Australian” or “older men” or “older women” or “older resident” or “older patient” or “senior citizen” or “late life” or “oldest old” or “aged 65” or “aged 70” or “aged 75” or “aged 80” or “aged 85”):ti,ab

7 ("systematic review" or "meta analysis" or "meta-analysis" or "scoping review" or “realist review” or “systematic integrative review” or “mapping review” or “critical review” or “qualitative review” or “meta synthesis” or “quantitative review” or "rapid review):ti,ab

8 #1 or #2

9 #3 or #4

10 #5 or #6

11 #7 and #8 and #9 and #10
