# Supplementary material for: Quality indicators for palliative care for older people: An umbrella review
Source: Palliat Med. 2025 Dec 29;40(3):284–96. doi: 10.1177/02692163251403422 (PMC12936153; doi:10.1177/02692163251403422)
Supplement: sj-docx-2-pmj-10.1177_02692163251403422 – Supplemental material for Quality indicators for palliative care for older people: An umbrella review [file sj-docx-2-pmj-10.1177_02692163251403422.docx]

**Supplementary File 2: Definitions of Essential elements for safe and high‑quality end‑of‑life care: National Consensus Statement**

Australian Commission on Safety and Quality in Health Care

Table S1. Care Processes: Essential elements and Definitions as adapted from the Australian Commission on Safety and Quality in Health Care (1)

| **Essential Element** | **Definition of element** |
| --- | --- |
| Recognising end of life | Routine screening and assessment to determine if a person is nearing the end of life. |
| Person‑centred communication and shared decision making | Ensuring that care is person-centred, which requires health care workers to support the person in their decision making, and communicate in a way that is responsive to the needs of the person. |
| Multidisciplinary collaboration and coordination of care | People benefit from the input of multidisciplinary teams, however, it is essential that care is coordinated across care providers and settings. |
| Comprehensive care | Those providing end of life care should comprehensively assess, monitor, and respond to the needs and condition of a person at the end of life. This care should be culturally safe, aligned with the expressed wishes of the person, and encompass medical treatment along with psychosocial, spiritual, cultural, religious and emotional support. |
| Responding to concerns | Where concerns are raised from the person, or their family/substitute decision maker, these should be responded to in a timely and appropriate manner. These concerns should be escalated when the support of external, specialised staff is needed. |

Table S2. Organisational processes: Essential elements and Definitions as adapted from the Australian Commission on Safety and Quality in Health Care (1)

| **Essential Element** | **Definition of element** |
| --- | --- |
| Leadership and governance | Services are required to use established systems and processes to deliver end of life care, which includes committed leadership, appropriate use of policies and processes, and the employment of appropriately trained and qualified staff to deliver end of life care. |
| Support, education and training | All staff caring for people at the end of life should have appropriate education and training on the policies, process and practices that affect end of life care. |
| Care setting | The care setting is an important aspect at the end of life, and should take into account the persons preferences, alongside the appropriateness of the setting which can include out of hours or unrestricted access to the palliative person, and access to private spaces for cultural practices, rituals, meetings or family gatherings. |
| Evaluation, audit and feedback | Deaths should be routinely reviewed by the healthcare service to determine the safety and quality of the person’s end of life care. |
| Systems to support high‑quality care | Healthcare organisations should systematise the approach to end of life care which can include the use of digital platforms, access to specialist palliative care and end of life medications, and appropriate mechanisms for documenting and sharing end of life/comprehensive care plans and advance care plans/directives. |

References

1. Australian Commission on Safety and Quality in Health Care. National Consensus Statement: Essential elements for safe and high‑quality end‑of‑life care. Sydney; 2023.
