# Supplementary material for: Quality indicators for palliative care for older people: An umbrella review
Source: Palliat Med. 2025 Dec 29;40(3):284–96. doi: 10.1177/02692163251403422 (PMC12936153; doi:10.1177/02692163251403422)
Supplement: sj-docx-3-pmj-10.1177_02692163251403422 – Supplemental material for Quality indicators for palliative care for older people: An umbrella review [file sj-docx-3-pmj-10.1177_02692163251403422.docx]

Supplementary File 3: Joanna Briggs Institute Critical Appraisal Instrument for Systematic Reviews and Research Synthesis Appraisal (1)

|  | Amador et al. (2) | Karimi-Dehkordi et al. (3) | Mitchell et al. (4) | Yorganci et al. (5) |
| --- | --- | --- | --- | --- |
| 1. Is the review question clearly and explicitly stated? | Y | Y | Y | Y |
| 1. Were the inclusion criteria appropriate for the review question? | Y | Y | Y | Y |
| 1. Was the search strategy appropriate? | Y | Y | Y | Y |
| 1. Were the sources and resources used to search for studies adequate? | Y | Y | Y | Y |
| 1. Were the criteria for appraising studies appropriate? | N/A | Y | N/A | N/A |
| 1. Was critical appraisal conducted by two or more reviewers independently? | N/A | No | N/A | N/A |
| 1. Were there methods to minimize errors in data extraction? | Y | Unclear | Y | Y |
| 1. Were the methods used to combine studies appropriate? | Y | Y | Y | Y |
| 1. Was the likelihood of publication bias assessed? | N/A | N/A | N/A | Y |
| 1. Were recommendations for policy and/or practice supported by the reported data? | Y | Y | Y | Y |
| 1. Were the specific directives for new research appropriate? | Y | Y | Y | Y |
| Overall appraisal | Include | Include | Include | Include |

References

1. Aromataris E, Lockwood C, Porritt K, Pilla B, Jordan Z. JBI Manual for Evidence Synthesis: JBI; 2024. Available from: <https://doi.org/10.46658/JBIMES-24-01>.

2. Amador S, Sampson EL, Goodman C, Robinson L. A systematic review and critical appraisal of quality indicators to assess optimal palliative care for older people with dementia. Palliative Medicine. 2019;33(4):415-29.

3. Karimi-Dehkordi M, Hanson HM, Kennedy M, Wagg A. Mapping Quality Indicators to Assess Older Adult Health and Care in Community-, Continuing-, and Acute-Care Settings: A Systematic Review of Reviews and Guidelines. Healthcare. 2024;12(14):1397.

4. Mitchell RJ, Wijekulasuriya S, du Preez J, Lystad R, Chauhan A, Harrison R, et al. Population-level quality indicators of end-of-life-care in an aged care setting: Rapid systematic review. Archives of Gerontology and Geriatrics. 2024;116.

5. Yorganci E, Sampson EL, Gillam J, Aworinde J, Leniz J, Williamson LE, et al. Quality indicators for dementia and older people nearing the end of life: A systematic review. Journal of the American Geriatrics Society. 2021;69(12):3650-60.
